# SIRT1 expression is associated with lymphangiogenesis, lymphovascular invasion and prognosis in pN0 esophageal squamous cell carcinoma

**DOI:** 10.1186/2045-3701-4-48

**Published:** 2014-08-26

**Authors:** Guan-qing Chen, Hui Tian, Wei-ming Yue, Lin Li, Shu-hai Li, Lei Qi, Cun Gao, Li-bo Si, Ming Lu, Fei Feng

**Affiliations:** Department of Thoracic Surgery, Qi Lu Hospital, Shandong University, Wen hua xi lu 107#, Jinan, 250012 Shandong Province China

**Keywords:** Esophageal squamous cell carcinoma, Lymphangiogenesis, Lymphatic metastasis, Prognosis, SIRT1

## Abstract

**Background:**

Sirtuin1 (SIRT1) is an NAD^+^-dependent type III histone deacetylase (HDAC). This research investigated the prevalence of SIRT1 protein expression and its prognostic influence with the aim of validating its potential role in lymphangiogenesis and lymphovascular invasion (LVI) in pN0 esophageal squamous cell carcinoma (ESCC).

**Methods:**

A total of 206 patients were enrolled in this retrospective study. SIRT1 and VEGF-C protein expression was detected by immunohistochemical staining. Peritumoral lymphatic microvessel density (LVD) and LVI were evaluated by immunostaining for D2-40. Statistical analysis was then preformed to investigate the relevance of SIRT1 expression and various clinicopathologic features and to examine the effect of SIRT1 on tumor-induced lymphangiogenesis, LVI and prognosis.

**Results:**

SIRT1 positive expression was identified in 95 cases in the nucleus and was significantly correlated with T status (*P* < 0.001), disease stage (*P* = 0.001), VEGF-C positive expression (*P* = 0.015), high LVD (*P* = 0.013) and positive LVI (*P* = 0.015). Patients with SIRT1 positive expression, high LVD and positive LVI had a significantly unfavorable 5-year disease free survival (*P* < 0.001, *P* = 0.030, and *P* < 0.001, respectively) and overall survival (*P* < 0.001, *P* = 0.017, and *P* < 0.001, respectively). However, based on multivariate Cox regression analysis, only SIRT1 positive expression and positive LVI were significant independent prognosticators of poor disease-free survival (*P* = 0.029 and 0.018, respectively) and overall survival (*P* = 0.045 and 0.031, respectively).

**Conclusions:**

SIRT1 positive expression was significantly associated with tumor progression, lymphangiogenesis, LVI and poor survival in pN0 ESCC patients. Our research shows a utilization of SIRT1 in prognosing poor survival and providing possible target for ESCC patients through inhibiting its lymphangiogenesis activity.

## Background

Esophageal cancer (EC) is one of the most common malignancies worldwide, and it ranks as the sixth major cause of cancer-related death [1]. Esophageal squamous cell carcinoma (ESCC) is the predominant subtype of this lethal disease, especially in China. Annually, approximately half of the newly diagnosed EC patients worldwide are Chinese, and nearly 90% of these patients have ESCC [2–4]. In the past few decades, with the progress in surgical techniques and the implementation of neoadjuvant therapy, the outcome of EC has been improved; however, the 5-year survival rate remains low, especially in patients with lymph node metastasis [5, 6]. However, even in patients without lymph node metastasis (pN0), some of them still develop metastasis and have poor prognosis after surgery [7]. Until now, efficient biomarkers for pN0 ESCC patients that could be useful for further risk classification have not been identified. Thus, identification of prognostic molecular markers for pN0 ESCC may help to identify patients with poor prognosis who would benefit from further clinical treatment, provide possible therapeutic targets and improve the long-term survival rate.

Protein acetylation plays an important role in cancer development and progression [8]. Sirtuin1 (SIRT1) is an NAD^+^-dependent type III histone deacetylase (HDAC) [9]. By diminishing the acetylation of histones and non-histone substrates, such as p53 and p73, SIRT1 participates in various signaling pathways related to aging, DNA repair, metabolic regulation, apoptosis, and proliferation [10–12]. Although much research has been performed on SIRT1, its role in tumorigenesis in specific cancers, even in the same cancer type, is still controversial [13]. It has been shown that SIRT1 positive expression is strongly associated with tumorigenesis and tumor progression in various cancer types, such as colorectal cancer [14], gastric carcinoma [15], prostate cancer [16], lung cancer [17], and breast cancer [18]. However, convincing evidence has also demonstrated the tumor suppressor function of SIRT1 [19, 20]. Until now, no studies have evaluated the correlation of SIRT1 with clinicopathologic characteristics and prognosis in ESCC.

Lymph metastasis is the dominant means by which ESCC disseminates systemically [21, 22], and it has been shown to be correlated with enhanced lymphangiogenesis and positive LVI in some cancer types [23, 24]. Nonetheless, data on lymphangiogenesis and LVI in ESCC are still rare. A recent report has indicated that VEGF-C, the primary mediator of lymphangiogenesis, is a downstream factor regulated by SIRT1 [25]. To the best of our knowledge, this is the first clinical study to examine the expression level of SIRT1 protein in pN0 ESCC patients and to elucidate the relationship between SIRT1 protein expression and various clinicopathologic features, tumor lymphangiogenesis, LVI and prognosis.

## Results

### Correlations among SIRT1, VEGF-C and clinicopathologic features

**Figure 1:**
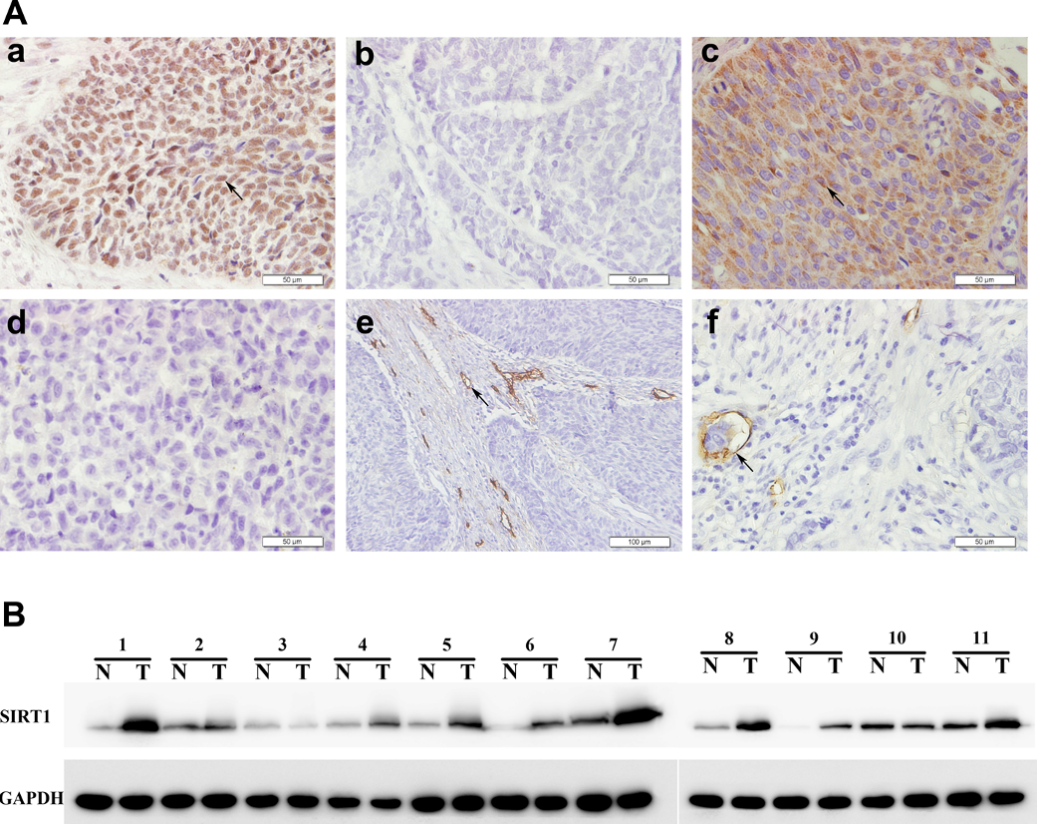
**SIRT1, VEGF-C and D2-40 expression in esophageal tissue samples. (A)** Immunohistochemical staining of SIRT1, VEGF-C protein, lymphatic microvessels and lymphovascular invasion in pN0 ESCC tissues: (a) SIRT1 positive expression; (b) SIRT1 negative expression (magnification × 400); (c) VEGF-C protein positive expression; (d) VEGF-C protein negative expression (magnification × 400); (e) lymphatic microvessel labeled with D2-40 in peritumoral stromal tissue (magnification × 200); (f) lymphovascular invasion highlighted by D2-40 (magnification × 400). **(B)** SIRT1 protein expression was evaluated by western blotting in ESCC tissues and paired noncancerous tissues.

SIRT1 positive expression was detected mainly in the nuclei, while VEGF-C was found in the cytoplasm (Figure 1A). Among the 206 patients, 95 (46.1%) showed SIRT1 positive expression, while 111 (53.9%) showed SIRT1 negative expression (Figure 1A). A total of 107 (51.9%) patients showed VEGF-C positive expression, while 99 (48.1%) showed VEGF-C negative expression (Figure 1A). Moreover, negative controls (PBS) showed no immunostaining. Correlations among SIRT1, VEGF-C and clinicopathologic characteristics are shown in Table 1.To further analyze the expression level of SIRT1 in ESCC, we performed western blot analysis in 11 fresh biopsies of paired primary tumor tissue and corresponding nontumorous tissue. As shown in Figure 1B, the results confirmed the overexpression of SIRT1 in ESCC tumor tissues when compared with paired nontumorous tissues.

**Table 1:** **Correlation of clinicopathologic characteristics with SIRT1 protein, VEGF-C protein, LVD and LVI**

Variables	SIRT1		***P***	VEGF-C		***P***	LVD		***P***	LVI		***P***
	Negative (n = 111)	Positive (n = 95)		Negative (n = 99)	Positive (n = 107)		Low (n = 123)	High (n = 83)		Negative (n = 135)	Positive (n = 71)	
Gender			0.975			0.516			0.171			0.897
Male	82	70		71	81		95	57		100	52	
Female	29	25		28	26		28	26		35	19	
Age (year)			0.697			0.786			0.842			0.327
<50	30	28		27	31		34	24		35	23	
≥50	81	67		72	76		89	59		100	48	
Weight loss (kg)			0.205			0.834			0.940			0.239
<2.5	67	49		55	61		69	47		80	36	
≥2.5	44	46		44	46		54	36		55	35	
Length of tumor (cm)			0.654			0.492			0.991			0.980
<3	45	43		45	43		53	35		58	30	
3-5	55	41		42	54		57	39		63	33	
>5	11	11		12	10		13	9		14	8	
Differentiation			0.070			0.125			0.294			0.027
Well	27	13		25	15		28	12		30	10	
Moderate	56	62		53	65		69	49		81	37	
Poor	28	20		21	27		26	22		24	24	
Tumor location			0.227			0.083			0.765			0.370
Upper	17	8		17	8		16	9		19	6	
Middle	56	57		49	64		65	48		70	43	
Lower	38	30		33	35		42	26		46	22	
T status			<0.001			0.025			0.439			0.036
T1	26	7		23	10		23	10		26	7	
T2	63	47		48	62		64	46		75	35	
T3	22	41		28	35		36	27		34	29	
Stage			0.001			0.001			0.069			0.039
I	30	13		31	12		32	11		35	8	
IIa	47	28		35	40		44	31		48	27	
IIb	34	54		33	55		47	41		52	36	

LVD: lymphatic microvessel density.

LVI: lymphovascular invasion.

SIRT1 positive expression was significantly correlated with T status (*P* < 0.001) and stage (*P* = 0.001). No other significant associations were observed between SIRT1 expression and age, gender, differentiation, weight loss, tumor location, or length of tumor (*P* > 0.05).

VEGF-C positive expression was significantly correlated with T status (*P* = 0.025) and stage (*P* = 0.001). However, no other clinicopathologic features were significantly associated with VEGF-C positive expression (*P* > 0.05).

### Correlations among lymphatic microvessel density (LVD), LVI and clinicopathologic features

Lymphatic vessels were detected by staining of D2-40, which is specifically expressed in the lymphatic endothelium but not in vascular endothelial cells (Figure 1A). Of the 206 tissues, 83 (40.3%) showed high LVD, and 71 (34.5%) showed positive LVI. There was no significant relationship between LVD and any clinicopathologic features (*P* > 0.05, Table 1). However, positive LVI was significantly correlated with differentiation (*P* = 0.027), T status (*P* = 0.036) and stage (*P* = 0.039). No other clinicopathologic features showed a significant association with positive LVI (*P* > 0.05).

### Correlations among SIRT1, VEGF-C, LVD and LVI

As shown in Table 2, SIRT1 positive expression was significantly correlated with VEGF-C positive expression (*P* = 0.015). Additionally, the LVD was greater in SIRT1-positive tissues and VEGF-C-positive tissues (*P* = 0.013 and 0.005, respectively). Positive LVI was also increased in SIRT1-positive tissues (*P* = 0.015), VEGF-C-positive tissues (*P* = 0.007) and tissues with high LVD (*P* = 0.027).

**Table 2:** **Correlations between SIRT1, VEGF-C, LVD and LVI**

Variables	SIRT1		***P***	VEGF-C		***P***	LVD		***P***	LVI		***P***
	Negative (n = 111)	Positive (n = 95)		Negative (n = 99)	Positive (n = 107)		Low (n = 123)	High (n = 83)		Negative (n = 135)	Positive (n = 71)	
VEGF-C			0.015			-						
Negative	62	37		-	-							
Positive	49	58		-	-							
LVD			0.013			0.005			-			
Low	75	48		69	54		-	-				
High	36	47		30	53		-	-				
LVI			0.015			0.007			0.027			
Negative	81	54		74	61		88	47		-	-	-
Positive	30	41		25	46		35	36		-	-	

LVD: lymphatic microvessel density.

LVI: lymphovascular invasion.

### Correlations among SIRT1, VEGF-C, LVD, LVI and tumor recurrence

**Figure 2:**
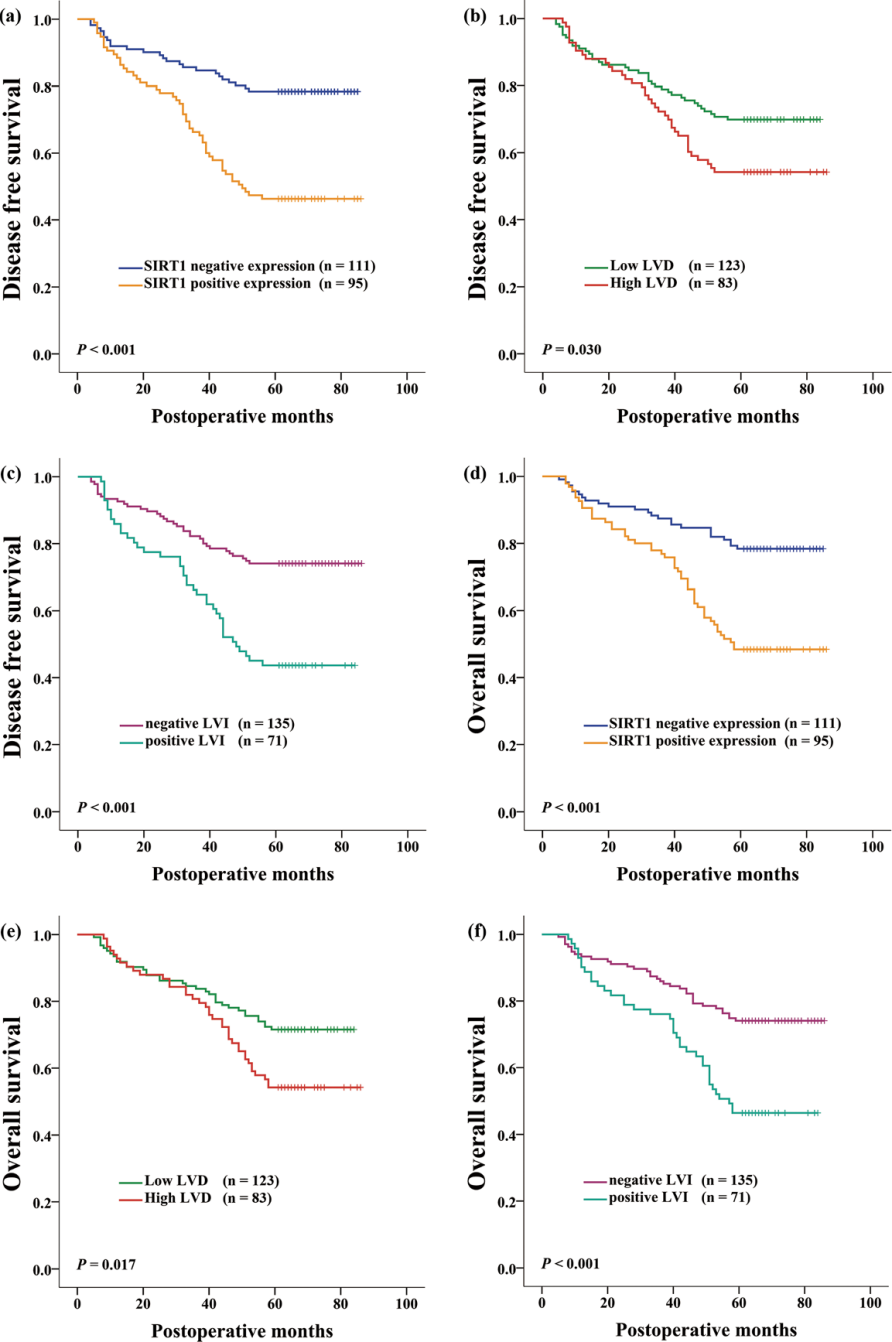
**Kaplan-Meier survival analysis of ESCC patients.** Disease-free survival stratified by SIRT1 expression **(a)**, LVD **(b)**, LVI **(c)**. Overall survival stratified by SIRT1 expression **(d)**, LVD **(e)**, LVI **(f)**.

Recurrence occurred in 75 (36.4%) patients during 5-year follow-up. A total of 19 patients were diagnosed with locoregional relapse, 34 patients were diagnosed with distant metastasis, and 22 patients were diagnosed with both locoregional relapse and distant metastasis. Overall, 51 (53.7%) of the 95 SIRT1-positive patients and 24 (21.6%) of the 111 SIRT1-negative patients suffered tumor relapse (*P* < 0.001, chi-square test). Tumor recurrence was observed in 44 (41.1%) of 107 VEGF-C-positive patients and 31 (31.3%) of 99 VEGF-C-negative patients (*P* = 0.144, chi-square test). In 83 cases with high LVD, 38 (45.8%) patients developed tumor relapse, and in 123 cases with low LVD, 37 (30.1%) patients developed tumor relapse (*P* = 0.022, chi-square test). A total of 40 (56.3%) of 71 patients with positive LVI and 35 (25.9%) of 135 patients with negative LVI suffered tumor relapse (*P* < 0.001, chi-square test). Kaplan-Meier curves of the 5-year disease-free survival (DFS) showed that SIRT1 positive expression (*P <* 0.001, Figure 2a), high LVD (*P* = 0.030, Figure 2b) and positive LVI (*P <* 0.001, Figure 2c) had significantly unfavorable prognostic influences. To objectively reflect the prognostic influence of the risk factors that were detected in univariate analysis, only significant risk factors were further tested by multivariate Cox regression analysis. However, based on multivariate analysis, only SIRT1 positive expression (*P* = 0.029) and positive LVI (*P* = 0.018) were independent prognostic factors for DFS (Table 3).

**Table 3:** **Univariate and multivariate analyses of prognostic variables**

Variables	Univariate analysis		Multivariate analysis			
	***P*** ^a^	***P*** ^b^	HR (95.0% CI)^a^	***P*** ^a^	HR (95.0% CI)^b^	***P*** ^b^
Age	0.928	0.832				
Gender	0.192	0.243				
Weight loss	0.573	0.578				
Length of tumor	0.685	0.623				
Tumor location	0.358	0.292				
Differentiation	0.017	0.023	1.302 (0.861-1.971)	0.211	1.271 (0.835-1.934)	0.262
T status	<0.001	<0.001	1.828 (1.176-2.842)	0.007	1.742 (1.116-2.720)	0.015
Stage	<0.001	<0.001	1.567 (1.032-2.381)	0.035	1.538 (1.010-2.342)	0.045
VEGF-C	0.172	0.258				
LVI	<0.001	<0.001	1.782 (1.106-2.872)	0.018	1.702 (1.050-2.757)	0.031
LVD	0.030	0.017	1.146 (0.720-1.823)	0.565	1.244 (0.777-1.991)	0.364
SIRT1	<0.001	<0.001	1.784 (1.061-3.001)	0.029	1.711 (1.013-2.890)	0.045

^**a**^Disease free survival;

^**b**^Overall survival;

CI confidence interval.

### Univariate and multivariate survival analyses

The overall 5-year survival rate was 64.6%, and 73 patients eventually died after the operation. We found that patients with SIRT1 positive expression had a significantly shorter 5-year overall survival (OS) than patients who lacked SIRT1 expression (48.4% vs 78.4%; *P* < 0.001, Figure 2d). High LVD was also significantly associated with worse 5-year OS compared with low LVD (54.2% vs 71.5%, *P* = 0.017, Figure 2e). In addition, positive LVI was also associated with a significantly shorter 5-year OS than negative LVI (46.5% vs 74.1%, *P* < 0.001, Figure 2f). There was no significant association between VEGF-C positive expression and poor 5-year OS (*P* = 0.258, Table 3). Furthermore, multivariate Cox regression analysis revealed that SIRT1 positive expression (*P* = 0.045) and positive LVI (*P* = 0.031) were independent prognosticators of poor OS (Table 3).

## Discussion

SIRT1 protein, which plays an important role in deacetylation, has been demonstrated to be involved in tumorigenesis and lymph node metastasis in various types of human cancers [14–17]. To the best of our knowledge, this is the first report examining the expression of SIRT1 protein in pN0 ESCC patients and the first demonstration of the relationship between SIRT1 protein and clinicopathologic features, lymphangiogenesis, LVI and prognosis. Our results indicate that SIRT1 protein may have a tumor-promoting function in ESCC patients, as its positive expression was significantly correlated with T status and stage.

Recent reports have indicated that deacetylation of FOXO-1 by SIRT1 could enhance VEGF-C transcription in the nuclei of prostate cancer cells and facilitate the growth of endothelial cells in mice [13, 25]. Thus, we hypothesized that SIRT1 may also enhance VEGF-C transcription in ESCC. According to our findings, SIRT1 positive expression in the nucleus was significantly correlated with VEGF-C positive expression, suggesting a possible role for SIRT1 in regulating VEGF-C expression in ESCC; however, these findings are preliminary. The specific molecular mechanism underlying this process is still not well understood and requires further elucidation in the future. It has been shown that VEGF-C is an essential factor in lymphangiogenesis [26] and LVD can significantly affect LVI [24]; thus, we also tested the correlation among VEGF-C, LVI and LVD and the possible relevance of SIRT1, LVI and LVD. As shown in Table 2, VEGF-C and LVI were significantly correlated with LVD, in accordance with previous reports [27, 28]. Additionally, our results showed a significantly high LVD and positive LVI in patients with SIRT1 positive expression, indicating an important role for SIRT1 in promoting tumor lymphangiogenesis and lymphatic metastasis.

Until now, studies on lymphangiogenesis and LVI in ESCC patients have been rare and contradictory, and there are no available data for pN0 ESCC patients. Previous studies have shown that ESCC patients with high LVD have a significantly worse prognosis [29], while Schoppmann [28] showed a significant prognostic impact in adenocarcinomas only. On the other hand, positive LVI has been shown to be a significant prognosticator for all types of esophageal cancers.

Our research demonstrated higher rates of tumor recurrence and worse OS in patients with high LVD, positive LVI and SIRT1 positive expression. However, although VEGF-C positive expression was significantly correlated with T status and stage, which agreed with previous research [30], VEGF-C positive expression was not prognostic for poor DFS or OS. These results may be due to the complexity of lymphangiogenesis regulation, the heterogeneity of the microenvironment of different cancer types and the activation of different signaling pathways, even in the same tumor type, at different stages. Univariate analysis revealed the value of LVD, LVI and SIRT1 in prognosing tumor relapse and poor OS. However, based on multivariate analysis, only SIRT1 positive expression and positive LVI remained as independent prognostic factors for both DFS and OS. Taken together, these results support the hypothesis that SIRT1 may promote tumor progression partially by induction of lymphangiogenesis and LVI.

It should be noted that the mechanisms of tumor lymphangiogenesis and lymphatic metastasis are exceptionally complex, and the exact role of SIRT1 protein in lymphangiogenesis and lymphatic metastasis remains to be further studied. Recent reports have indicated that tumor-induced lymphangiogenesis may not only supply a number of draining tubes for cancer cell metastasis but may also regulate host immune status and reflect changes in the tissue microenvironment [31]. Debates regarding the function of peritumoral and intratumoral lymphatic vessels are ongoing. In esophageal cancer, this contradiction is mainly due to the different definition of intratumoral lymphatic vessels. Lymphatic vessels are always found in peritumoral stromal tissue [28].

The debate regarding the function of SIRT1 in tumorigenesis is ongoing. Because SIRT1 participates in various signaling pathways [10–12], it may function differently in each case. Tumorigenesis is an extremely complex process involving numerous signaling pathways; thus, the importance of SIRT1 in specific tumors may also vary. Further studies will be undertaken in the future, and autologous normal tissues may also be used to help us further understand the role of SIRT1 in tumorigenesis in ESCC patients.

## Conclusion

In conclusion, our study revealed the potential role of SIRT1 protein in the progression, lymphangiogenesis and lymphatic metastasis of pN0 ESCC. In addition, we showed that positive expression of SIRT1 protein was significantly associated with unfavorable prognosis, indicating that SIRT1 protein may be useful in predicting prognosis and could represent a novel therapeutic target for ESCC patients.

## Materials and methods

### Patients

Between January 2004 and December 2007, 206 patients who were diagnosed with pN0 ESCC by pathological examination after operation at the Department of Thoracic Surgery, Qilu Hospital, were enrolled in this retrospective study. For western blotting analysis, 11 matched pairs of freshly biopsied tumor tissues and corresponding nontumorous tissues were collected immediately after resection between October 2013 and November 2013 in our department and stored at −80°C. All patients underwent esophagectomy and esophagogastric anastomosis with regional lymph node dissection. Of all the 206 patients, 2556 lymph nodes were dissected (mean of 12.4 per case, ranging from 10 to 19). Information on the patients’ follow-up and clinicopathologic features were collected. None of patients received chemotherapy or radiotherapy before surgery. This research was approved by the Ethics Committee of Qilu Hospital.

There were 152 men and 54 women, ranging in age from 40 to 78 years (mean 58.57 ± 11.65 years). According to the TNM classification system of the 7th edition of the AJCC Cancer Staging criteria, tumor stage was ascertained after surgery by expert pathologists. The clinicopathological characteristics of the patients are shown in Table 4.

**Table 4:** **Clinicopathological characteristics of the patients**

Variables	No. of patients
Gender	
Male	152 (73.8%)
Female	54 (26.2%)
Age (year)	
<50	58 (28.2%)
≥50	148 (71.8%)
Weight loss (kg)	
<2.5	116 (56.3%)
≥2.5	90 (43.7%)
Length of tumor (cm)	
<3	88 (42.7%)
3-5	96 (46.6%)
>5	22 (10.7%)
Differentiation	
Well	40 (19.4%)
Moderate	118 (57.3%)
Poor	48 (23.3%)
Tumor location	
Upper	25 (12.1%)
Middle	113 (54.9%)
Lower	68 (33.0%)
T status	
T1	33 (16.0%)
T2	110 (53.4%)
T3	63 (30.6%)
Stage	
I	43 (20.9%)
IIa	75 (36.4%)
IIb	88 (42.7%)
SIRT1	
Negative	111 (53.9%)
Positive	95 (46.1%)
VEGF-C	
Negative	99 (48.1%)
Positive	107 (51.9%)
LVD	
Low	123 (59.7%)
High	83 (40.3%)
LVI	
Negative	135 (65.6%)
Positive	71 (34.5%)

### Follow-up

Follow-up began on the day of hospital discharge. Patients were instructed to report to the outpatient clinic for follow-up evaluation once every 3 to 6 months and annually after the fifth year. Each evaluation included a physical and blood examination. Barium esophagram and chest radiography were performed every 3 months, and CT of the thorax and ultrasound were performed every 6 months in the first five years and once a year from then on. Fiberesophagoscopy and other specific procedures, such as MRI and emission computed tomography (ECT), were also preformed if necessary. Relapse was determined by pathological or radiological examination. Follow-up ended in March 2013, and it ranged from 5 to 86 months for all patients (average 55.9 months).

### Immunohistochemical staining

Immunohistochemistry was used to evaluate the expression of SIRT1, D2-40 and VEGF-C. Formalin-fixed and paraffin-embedded 4-μm thick tumor tissue slices were dewaxed and rehydrated before antigen retrieval. The microwave antigen retrieval method was then utilized, and the slides were immersed in EDTA antigen retrieval solution (pH 9.0) for 15 min. Subsequently, we added 3% hydrogen peroxide to the slides to inhibit endogenous peroxidase activity. Subsequently, SIRT1 (1:150; Abcam, Cambridge, UK), D2-40 (FLEX Ready-to-Use; Dako, Glostrup Denmark), and VEGF-C (1:100; Zhongshan Biotech, Beijing China) were applied to the sections that were later incubated at 4°C overnight (the incubation time of D2-40 was 25 minutes at room temperature according to the manufacturer’s recommendation). On the second day, biotinylated antibody and streptavidin-peroxidase reagent (Zhongshan Biotech, Beijing China) were successively applied for 15 min each at 37°C. Finally, 3,3’-diaminobenzidine tetrahydrochloride (DAB) was used for visualization, and hematoxylin was added as a counterstain.

The positive controls were human non-small cell lung cancer tissues expressing SIRT1 and VEGF-C protein. Sections that were incubated with PBS instead of primary antibodies were used as negative controls. Both the positive and negative controls were used to evaluate the reliability of staining and exclude nonspecific reactions.

### Western blotting analysis

Total proteins extracted from fresh tissues were prepared in radio immunoprecipitation assay (RIPA) buffer (Beyotime, Jiangsu, China) including complete protease inhibitor cocktail (Roche Applied Science, Mannheim, Germany). Total proteins were separated by 10% SDS-PAGE and then transferred to PVDF membranes. The membranes were blocked with 5% skim milk in Tris-buffered saline with 0.1% Tween-20 (TBST) for 1 h at room temperature and then incubated with anti-SIRT1 (1:1000; Abcam, Cambridge, UK) or anti-GAPDH (1:1000; Abcam, Cambridge, UK) antibodies overnight at 4°C. After incubation with horseradish peroxidase-conjugated anti-rabbit secondary antibodies for 1 h, proteins were detected using enhanced chemiluminescence (Millipore, Billerica, MA).

### Evaluation

The expression levels of SIRT1 and VEGF-C protein were calculated utilizing a semiquantitative scoring system. The staining score was classified as 0 (negative staining), 1 (weak staining), 2 (moderate staining) and 3 (strong staining). The quantity score, which represented the percentage of cancer cells that were positively stained, was calculated as follows: 0 (0-5%), 1 (6-25%), 2 (26-50%), 3 (51-75%), and 4 (≥76%). By multiplying the staining score by the quantity score of each slide, the final semiquantitative score was obtained (ranging from 0 to 12). Scores that ranged from 4–12 were considered to represent positive expression [16, 32].

Lymphatic microvessel density (LVD) was measured by quantifying vessels stained with D2-40. Three hot spots (the largest vessel density area stained) were first recognized at low power (×100), and then vessels were counted at high magnification (×200). The average number of positive vessels in six high-power areas (counted by two investigators) for each slide represents the LVD value. Tumors were categorized as High LVD and Low LVD according to the average LVD (16.07 ± 5.748 microvessels per × 200 magnification field (range from 0–29)). An LVD < 17 or ≥ 17 was designated as Low LVD or High LVD, respectively [33]. D2-40-stained lymphatic vessels containing at least one tumor cell were defined as Positive LVI [34].

### Statistical analysis

The SPSS software package (18.0; SPSS, Chicago, IL, USA) was used for statistical analysis. Correlations among SIRT1, VEGF-C, LVD, LVI and various clinicopathologic characteristics were compared using the chi-square test. Survival curves were constructed using the Kaplan-Meier method, and the significance of differences in the survival of subgroups was examined with the log rank test. Independent prognostic factors were determined by multivariate Cox regression analysis. *P* values less than 0.05 were considered significant.

## Consent

Written informed consent was obtained from the patient for the publication of this report and any accompanying images.
